# Beyond the Solid Solution: Ordered Enantiomerically Unbalanced Packing in Surface‐Confined Tetrahelicene Monolayers

**DOI:** 10.1002/chir.70106

**Published:** 2026-04-29

**Authors:** Aleksandra Cebrat, Kevin Martin, Pingo Mutombo, Manfred Parschau, Narcis Avarvari, Karl‐Heinz Ernst

**Affiliations:** ^1^ Empa Swiss Federal Laboratories for Materials Science and Technology Dübendorf Switzerland; ^2^ Univ Angers, CNRS MOLTECH‐Anjou, SFR MATRIX Angers France; ^3^ Nanosurf Lab Institute of Physics of the Czech Academy of Sciences Prague Czech Republic; ^4^ Department of Refining and Petrochemistry, Faculty of Oil, Gas and Renewable Energies University of Kinshasa Kinshasa Democratic Republic of the Congo

**Keywords:** chiral crystallization, conglomerate, helicenes, scanning tunneling microscopy, solid solution, surface science

## Abstract

The two‐dimensional crystallization and on‐surface chemistry of chiral polycyclic aromatic hydrocarbons provide access to surface‐confined symmetry breaking and novel covalently bonded nanostructures. Here, we investigate the self‐assembly of racemic 2,3‐dicarbonitrile tetrahelicene on Ag(111). Using low‐temperature scanning tunneling microscopy in combination with density functional theory calculations, we resolve the evolution of molecular organization as a function of coverage. After room‐temperature deposition at submonolayer coverage, the tetrahelicene moiety spontaneously resolves into extended homochiral Kagomé domains upon cooling. The resulting monolayer consists of hexameric dimer motifs arranged in a porous network. These mirror‐related supramolecular enantiomorphs are characteristic of two‐dimensional conglomerate crystallization. As the surface coverage increases, the system undergoes a transition toward denser packing. At saturation, compact domains form that retains long‐range lattice order while exhibiting a regular yet enantiomerically unbalanced distribution of both handednesses. The nonrandom incorporation of enantiomers distinguishes this phase from a racemic solid solution and instead indicates a compositionally biased, structurally ordered mixed phase.

## Graphical abstract

Racemic dicyanotetrahelicene exhibits coverage‐dependent self‐assembly on Ag(111), transitioning from spontaneous chiral resolution at low coverage to a dense mixed phase at monolayer saturation. While submonolayer deposition produces extended homochiral domains, full coverage yields a compact, long‐range ordered lattice with an enantiomerically unbalanced distribution of both enantiomers.

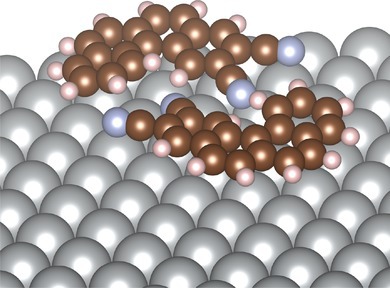

## 1 Introduction

As fundamental concept permeating the natural sciences [1, 2], chirality is particularly evident in crystallization processes [3, 4, 5], which have long played a central role in elucidating molecular structures and continue to serve as a cornerstone for the production of enantiopure pharmaceuticals and fragrances [6]. When a racemic mixture crystallizes, several outcomes are possible: the enantiomers may separate into enantiopure but oppositely handed crystal conglomerates, co‐crystallize within the same unit cell to form racemic compounds, or in rare cases are distributed randomly throughout the lattice as a solid solution [7, 8]. Despite of almost 180 years of experimental observation [9], reliably predicting which crystallization pathway a given chiral system will follow remains a major challenge.

To unravel the principles governing chiral crystallization, simplified and highly controllable model systems are essential. In this context, studies of the aggregation of chiral molecules on well‐defined surfaces provide a powerful approach to probing chirality at the microscopic level, which remains difficult to access in conventional crystallization due to the limited structural information available for crystal nuclei in solution or melts. Because heterogeneous nucleation is generally favored over homogeneous nucleation, the structure of the surface can strongly influence chiral discrimination and, consequently, the outcome of crystallization, including optical resolution. Investigating the nucleation and growth of chiral molecules on well‐defined surfaces therefore offers a controlled platform to elucidate the mechanisms underlying conglomerate and racemate formation. Beyond crystallization, such studies also provide insight into how chiral information propagates from individual molecules into extended supramolecular architectures, with implications for technologically relevant systems such as liquid crystals and heterogeneous enantioselective catalysis. In particular, scanning tunneling microscopy (STM) enables real‐space visualization of chiral recognition and assembly processes with submolecular resolution, offering direct insight into the mechanisms governing chiral organization in reduced‐dimensional systems [10, 11, 12, 13, 14].

Helicenes constitute a prominent family of polycyclic aromatic compounds distinguished by the intrinsic chirality that arises from the *ortho*‐fused arrangement of aromatic rings along a helical backbone [15, 16, 17]. Their rigid, nonplanar architecture endows them with pronounced chiroptical responses [18], making helicenes key molecular platforms for fundamental studies and applications in chiral optoelectronics. In particular, their well‐defined helicity has enabled advances in areas such as chiral photonics [19], circularly polarized light emission [20, 21, 22], and the chirality‐induced spin selectivity (CISS) effect [23, 24], where molecular handedness governs spin‐dependent electron transport, even at the single molecule level [25, 26]. Particularly noteworthy in this context is the magneto‐enantioselective adsorption of heptahelicene on ferromagnetic surfaces [27].

The surface‐confined self‐assembly and crystallization of helicenes have been extensively investigated by STM [28, 29], revealing a rich variety of two‐dimensional (2D) chiral ordering phenomena that depend sensitively on substrate, coverage, and functionalization. On Cu(100), for example, racemic heptahelicene ([7]H) forms 2D conglomerate phases in which enantiopure quadruplets persist up to monolayer coverage [30]. A closely related behavior has been reported for 5‐amino[6]helicene on the same surface [31], although alloying Cu(100) with tin induces a transition to racemate formation [32].

On Ag(100), [7]H undergoes a coverage‐dependent transition from conglomerate assemblies at low coverage to racemic zigzag rows at higher coverage [33]. An analogous behavior was observed for trioxa[11]helicene on Ag(100) [34], whereas, on Cu(100), this molecule aggregates into conglomerate domains [35]. Nucleation in the second layer of pentahelicene as well as [7]H was found to induce a transition from racemate to conglomerate in the first layer [36, 37].

A recent study described a rare example of a solid‐solution phase in two‐dimensional benzene‐1,3,5‐tris (tetrahelicene) crystals. On Ag(111), the self‐assembly process is driven by entropy maximization, and the proportion of the minority enantiomer within the hexagonally ordered monolayer scales with the density of topological defects [38].

Functionalization further modulates surface crystallization: cyano‐substituted [7]H assembles into 2D conglomerates on Cu(111) [39], while bromo‐, benzo‐, and S‐acetylthiolate‐functionalized derivatives form zigzag racemate structures [40]. Although such racemic zigzag arrangements remain chiral at the surface level, even a slight enantiomeric excess can suppress one surface enantiomorph through amplification of stereochemical interactions at domain boundaries [41].

Hydroxyl groups, on the other hand, induce the switch from a perpendicular surface orientation to a parallel surface alignment of the helical axis upon thermal activation [42].

**FIGURE 1:**
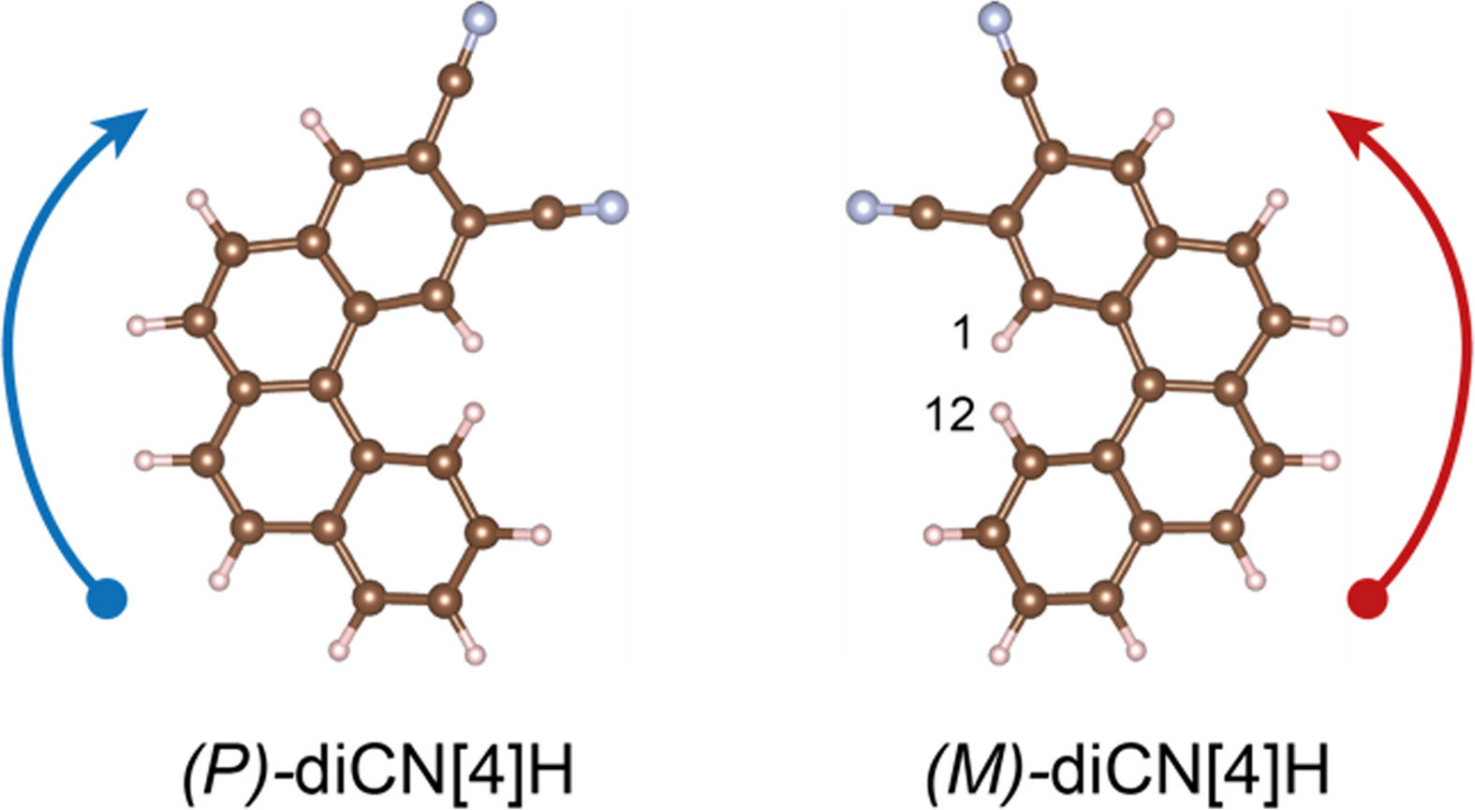
Enantiomers of 2,3‐dicyano tetrahelicene (diCN[4]H). Helicity is caused by steric overcrowding by hydrogen atoms at carbons 1 and 12 (H^12^ being above the projection plane). Arrows indicate the clockwise and counterclockwise helicity of (*P*)*‐* and (*M*) enantiomers.

Here, the self‐assembly of racemic 2,3‐dicarbonitrile tetrahelicene (diCN[4]H, Figure 1) on Ag(111) is investigated, with particular emphasis on the influence of surface coverage on two‐dimensional crystallization. With increasing lateral density, the system evolves from a two‐dimensional conglomerate to an ordered yet enantiomerically unbalanced phase.

## 2 Materials and Methods

### 2.1 Experimental

Experiments were performed under ultrahigh vacuum (base pressure < 1 × 10^−10^ mbar) using a home‐built low‐temperature scanning tunneling microscope. Atomically clean Ag(111) surfaces were prepared by repeated sputtering and annealing cycles. Molecules of racemic diCN[4]H were deposited by thermal sublimation (135°C) onto Ag(111) held at room temperature. Coverage was varied from ~0.2 monolayers (ML) to nominal monolayer saturation (~1.0 ML). A monolayer coverage (1 ML) is here defined by a full layer of flat‐lying molecules, as determined by STM. After deposition, samples were cooled to 7 K for STM imaging. Electrochemically etched tungsten tips were conditioned by gentle indentation into Ag to achieve Ag‐terminated apices. STM images were acquired in constant‐current mode with the bias voltage applied to the sample. The results presented herein are representative of the analysis of ca. 40 independent STM images acquired from 13 separate preparations.

### 2.2 Theoretical Analyses

DFT calculations were performed using the FHI‐aims code to find the adsorption geometry of diCN[4]H molecules on Ag(111) surface [43]. The calculations were carried out at the GGA‐PBE level, including the Tkatchenko−Scheffler treatment of the van der Waals interactions [44]. Relativistic effects were taken into account using the scaled zeroth‐order regular approximation [45]. The structural relaxation of single molecules, homochiral dimers and heterochiral dimers on the surface was performed using a 10 × 10 three‐layered slab. Note that all the atoms of the slab were relaxed except the bottom Ag layer. The calculations were considered converged when the total energy difference between consecutive steps and the remaining atomic forces were below 10^−6^ eV and 10^−2^ eV/Å, respectively. A single Γ‐point was used for the integration in the Brillouin zone.

### 2.3 Synthesis of Benzo[c]phenanthrene‐2,3‐Dicarbonitrile

All reagents and chemicals from commercial sources were used without further purification. Solvents were dried and purified using standard techniques. Column chromatography was performed with analytical‐grade solvents using Aldrich silica gel (technical grade, pore size 60 Å, 230–400 mesh particle size). Flexible plates ALUGRAM Xtra SIL G UV254 from MACHEREY‐NAGEL were used for TLC. Compounds were detected by UV irradiation (Bioblock Scientific) or staining with iodine, unless otherwise stated.

The new helicene derivative benzo[c]phenanthrene‐2,3‐dicarbonitrile was synthesized from the previously reported 2,3‐dibromobenzo[c]phenanthrene (**1**) [46], by substitution of the bromine atoms with cyano groups through the Rosenmund–von Braun reaction, following a published protocol [47]. As such, **1** was dissolved (156 mg, 404 μmol, 1 equiv.) together with CuCN (144.8 mg, 1.62 mmol, 4 equiv.) in DMF (10 mL). This solution was stirred for 16 h at reflux. Then, the organic layer was extracted with CH_2_Cl_2_, washed with H_2_O, brine, dried over MgSO_4_ and concentrated under vacuum. The crude was purified by chromatography over silica gel column (petroleum ether/CH_2_Cl_2_, 4:6, Rf = 0.3, Dragendorff TLC dips reagent was used). 25 mg (22% yield) of diCN[4]H were obtained as a white powder after evaporation of solvents.

Considering the quantities involved to perform this reaction, no specific treatment of the waste or unreacted copper cyanide was conducted. The solid was always handled under a ventilated fume hood while wearing the correct Personal Protective Equipment (PPE). The waste container was specifically labeled as containing cyanide.

^**1**^ **H NMR** (300 MHz, Chloroform‐*d*) δ 9.55 (s, 1H), 8.86 (d, *J* = 8.4 Hz, 1H), 8.49 (s, 1H), 8.17–8.06 (m, 3H), 7.99 (d, *J* = 8.6 Hz, 1H), 7.90 (d, *J* = 8.7 Hz, 1H), 7.86–7.73 (m, 2H).

^**13**^ **C NMR** (76 MHz, CDCl_3_) δ 135.45, 135.37, 134.99, 133.92, 133.62, 132.44, 132.02, 131.25, 130.65, 129.36, 129.22, 128.02, 127.53, 127.18, 126.46, 126.32, 110.55, 110.26.

**MS (EI+) m/z (**‐CN**) =** 252.8.

**NMR spectra** were recorded with a Bruker AVANCE III 300 (^1^H, 300 MHz and ^13^C, 76 MHz, ^19^F, 283 MHz, ^31^P, 122 MHz) and Bruker AVANCE DRX 500 (^1^H, 500 MHz and ^13^C, 125 MHz). Chemical shifts are given in ppm relative to tetramethylsilane TMS and coupling constants *J* in Hz. Residual nondeuterated solvent was used as an internal standard.

Matrix‐assisted laser desorption/ionization was performed on **MALDI‐TOF** MS BIFLEX III Bruker Daltonics spectrometer using dithranol, DCTB, or α‐terthiophene as matrix.

## 3 Results and Discussion

### 3.1 Low‐Coverage Self‐Assembly

**FIGURE 2:**
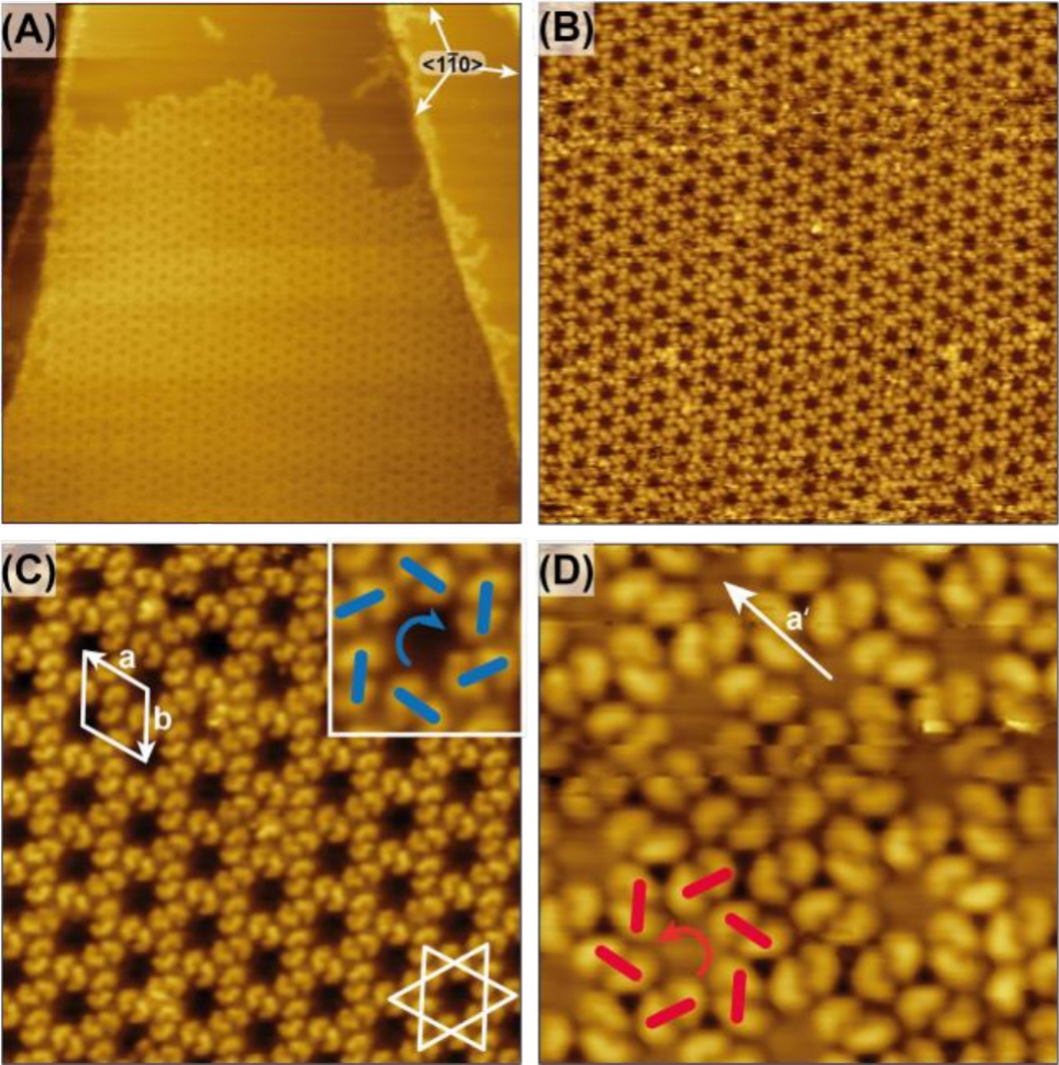
STM images of the spontaneous resolution of diCN[4]H at low coverage deposited onto Ag(111) at RT. (A) STM image (100 nm × 100 nm, *U* = 300 mV, *I* = 360 pA) of a self‐assembled island of the honeycomb structure. The high symmetry directions of the Ag(111) surface are indicated as arrows. (B) STM image (50 nm × 50 nm, *I* = 160 pA, *U* = 1.5 V) of an area covered with the honeycomb pattern. (C) STM image (20 nm × 20 nm, *I* = 200 pA, *U* = 350 mV) revealing a Kagomé lattice structure with diCN[4]H dimers as building blocks arranged in a clockwise fashion (blue arrow in inset). The unit cell, indicated as white rhombus, contains four molecules. (D) STM image (10 nm × 10 nm, *I* = 200 pA, *U* = 350 mV) of a domain with diCN[4]H dimers arranged in a counterclockwise fashion (red circular arrow). One lattice vector (**a′**, indicated by a white arrow) is tilt with respect to the corresponding lattice vector **a** of the mirror domain shown in (C) by 12°.

Following room‐temperature (RT) deposition at submonolayer coverages (20%–70% of a saturated monolayer, *θ* = 0.2–0.7 ML), diCN[4]H forms extended islands exhibiting a well‐defined honeycomb pattern across the Ag(111) terraces (Figure 2). The absence of isolated molecules indicates high surface mobility during deposition, enabling efficient two‐dimensional nucleation and growth. High‐resolution STM images reveal that the honeycomb lattice is composed of hexameric motifs, each formed by 12 molecules arranged around a central hollow cavity (Figure 2). The molecules within the hexameric motifs exhibit a comma‐shaped STM contrast and are arranged as six dimers. These dimers, in turn, constitute actually a Kagomé lattice (Figure 2C).

Each domain exhibits a well‐defined supramolecular chirality. First, the hexameric dimer arrangement adopts either a clockwise (Figure 2C) or counterclockwise pinwheel orientation (Figure 2D). Second, the enantiomorphous domains are rotated in opposite directions relative to the < 1 $\bar{1}$ 0 > high‐symmetry direction of the Ag(111) surface. The lattice vectors of domains with opposite handedness (white arrows in Figure 2C,D, labeled **a** and **a′**) enclose an angle of 12°.

The occurrence of mirror‐related domains composed of a single molecular handedness would demonstrate spontaneous resolution of the racemic adsorbate into a two‐dimensional conglomerate. Submolecular resolution reveals for most of the molecules in Figure 2C that one terminal “tail” of each molecule appears slightly brighter, suggesting a uniform handedness within a given domain. However, for surface‐confined tetrahelicenes, height differences between the terminal benzene rings are subtle, if present at all. Hence, reliable assignment of the absolute molecular handedness requires well‐defined STM tip conditions [38, 48, 49]. Moreover, the functionalization with cyano groups at one terminus is expected to further modify the STM contrast. Consequently, while homochirality can be concluded, the absolute handedness of the enantiomers within a single domain cannot be determined unambiguously by STM alone.

**FIGURE 3:**
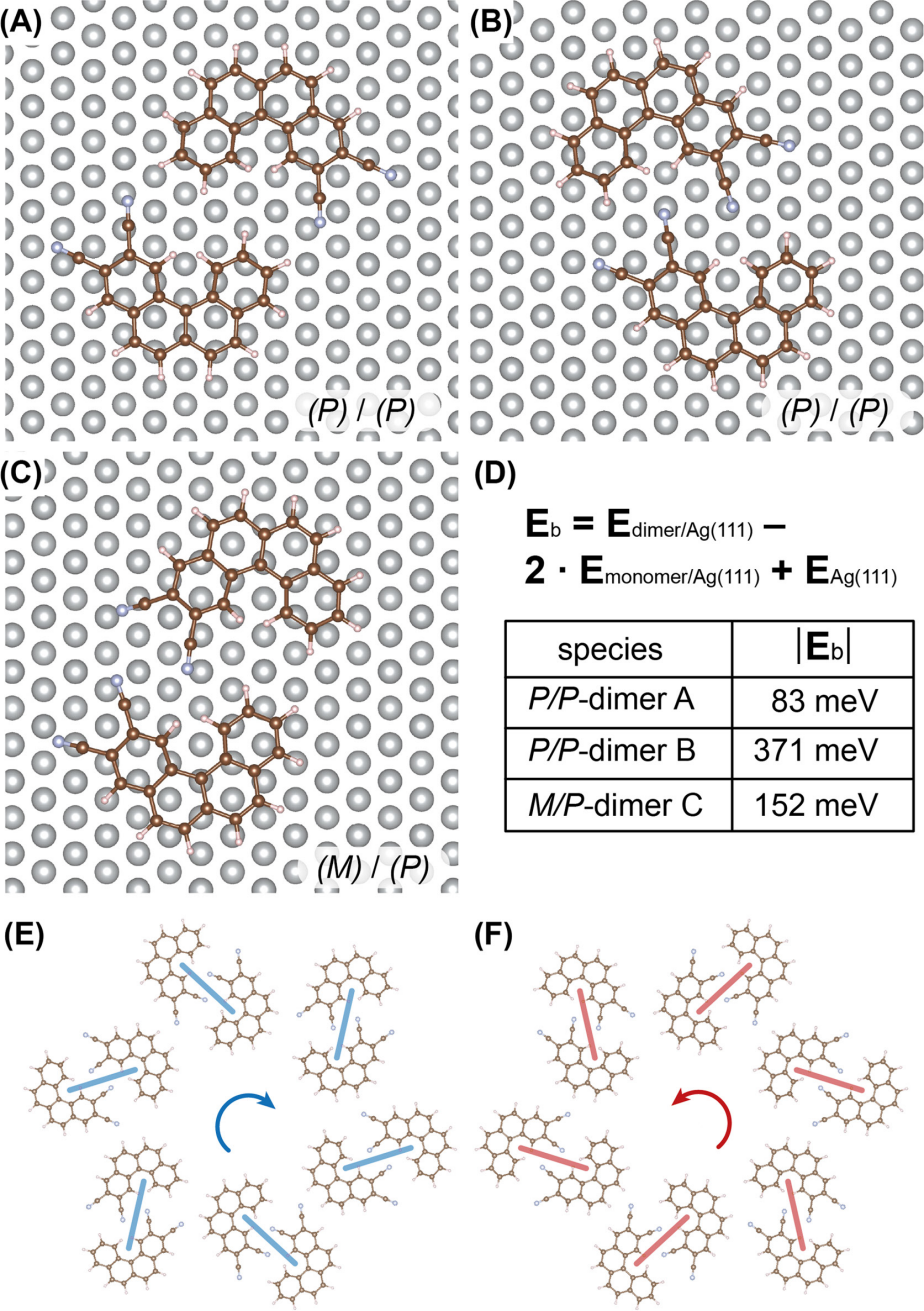
DFT modeling of homo‐ and heterochiral noncovalent dimers of diCN[4]H. (A) Hydrogen‐bonded head‐to‐tail configuration. (B) Configuration with cyano groups in parallel arrangement. (C) Low‐energy heterochiral dimer. (D) Interdimer binding energies of the three low‐energy results displayed in A–C. The homochiral dimer shown in (B) has the strongest intermolecular interaction. (E) Clockwise hexameric motif of lowest‐energy (*M,M*) dimers. (F) Counterclockwise hexameric motif of lowest‐energy (*P,P*) dimers.

To rationalize the occurrence of homochiral domains, STM observations were complemented by DFT calculations of dimer adsorption geometries. Figure 3 shows three examples of dimer formation. Several initial adsorption geometries were considered and fully relaxed. Among the low‐energy configurations, two homochiral arrangements were found to be particularly favorable. In the hydrogen‐bonded head‐to‐tail configuration (Figure 3A), intermolecular stabilization arises from complementary C–H···N interactions between the cyano substituent of one molecule and the terminal region of the adjacent helicene. A second low‐energy homochiral motif (Figure 3B) features, in addition to C–H···N hydrogen bonding, approximately parallel alignment of the cyano groups, optimizing dipolar and dispersive interactions. In contrast, the lowest‐energy heterochiral dimer (Figure 3C) exhibits a distinct packing geometry with reduced overall stabilization. Comparison of the calculated interdimer binding energies (Figure 3D) reveals that the homochiral dimer arrangement of Figure 3B is energetically preferred over the heterochiral configuration, providing a microscopic driving force for spontaneous resolution at low coverage.

On the basis of the most stable homochiral dimer motif, larger supramolecular assemblies were constructed. The optimized (*M,M*) dimers assemble into a clockwise hexameric arrangement (Figure 3E), whereas the mirror‐related (*P,P*) dimers form a counterclockwise motif (Figure 3F). These hexameric units reproduce the building blocks observed in the STM‐derived Kagomé lattice and directly account for the emergence of extended homochiral domains. The DFT results thus support the experimental finding that chiral recognition at the dimer level governs the formation of two‐dimensional conglomerate phases at low surface coverage.

### 3.2 High Coverage and Densification

**FIGURE 4:**
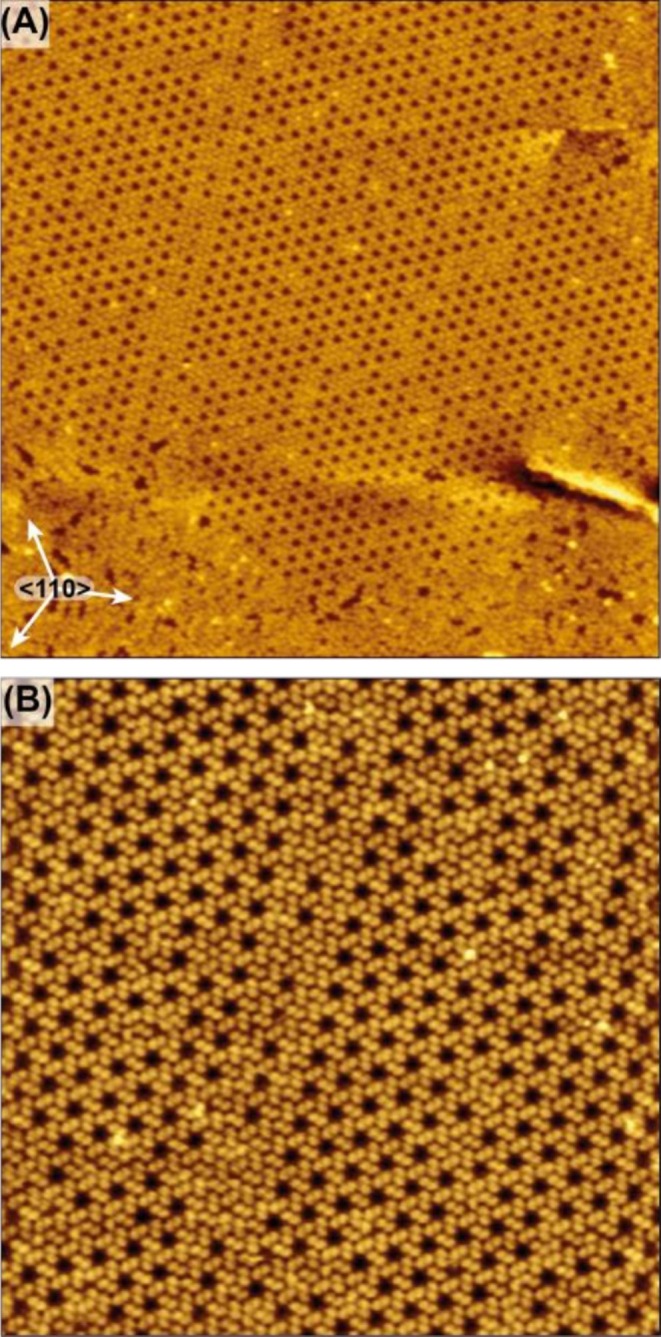
(A) STM image (100 nm × 100 nm, *I* = 7 nA, *U* = 1.3 V) of a honeycomb domain next to a disordered area (bottom) at intermediate monolayer coverage (*θ* ~ 0.8 ML). The high symmetry directions of the Ag(111) surface are indicated as arrows. (B) The STM image (50 nm × 50 nm, *I* = 8.2 nA, *U* = 1.3 V) taken at a coverage of *θ* ~ 0.8 ML shows besides honeycombs patches densely packed with dimers.

Increasing the surface coverage beyond the regime of extended honeycomb domains induces a pronounced structural transformation. At intermediate coverage (*θ* ~ 0.8 ML), the porous Kagomé network progressively destabilizes and coexists with disordered regions and short molecular rows (Figure 4). The reduction of pore density reflects the increasing importance of packing efficiency over the open hexameric motif that dominates at low coverage. Concomitantly, domain boundaries become more frequent, indicating competition between the low‐density conglomerate phase and emerging compact arrangements.

**FIGURE 5:**
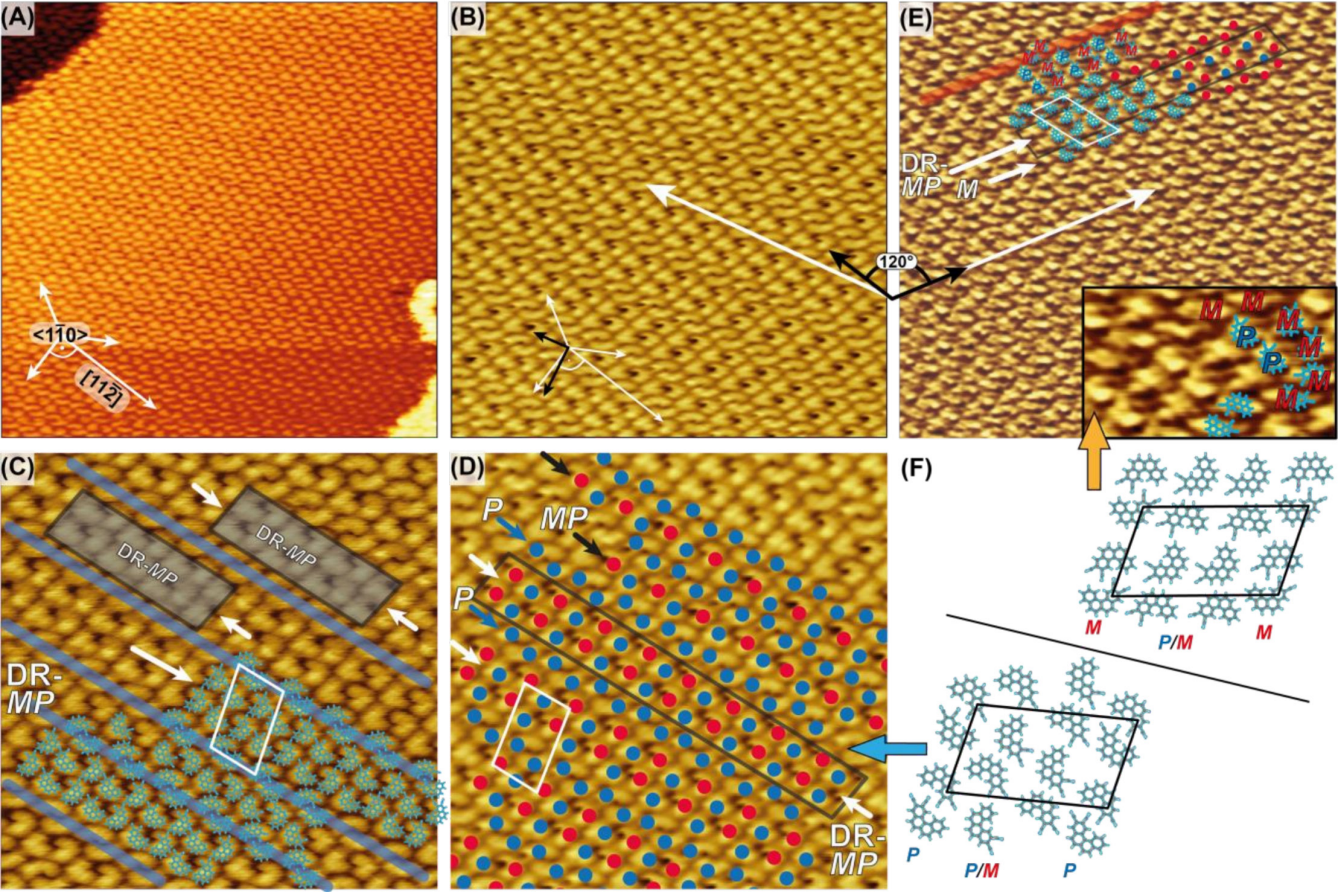
STM images taken at full monolayer coverage. (A) STM image of a fully covered substrate terrace (50 nm × 50 nm, *I* = 2 nA, *U* = 200 mV). The high symmetry directions of the Ag(111) surface are indicated as white arrows. (B) STM image (30 nm × 30 nm, *I* = 2 nA, *U* = 150 mV) of the well‐ordered monolayer. Two small black arrows indicate the unit cell vectors in direct comparison with the Ag(111) surface lattice directions (small white arrows as in (A)). One adsorbate lattice vector, placed over a homochiral dimer row, is indicated by a large white arrow. (C) STM image (18 nm × 18 nm, *I* = 2 nA, *U* = 130 mV; manually drift‐corrected), partially superimposed with molecular models of both enantiomers. The two‐dimensional lattice is composed of racemic double rows (**DR‐*MP***, highlighted by gray semitransparent boxes and indicated by white arrows) and homochiral dimer rows (marked by blue semitransparent lines). The unit cell is outlined by a white parallelogram. (D) Area (18 nm × 18 nm; manually drift‐corrected) extracted from the STM image shown in (B). Enantiomers are color‐coded with blue and red dots. Racemic double rows (**DR‐*MP***) are indicated by white arrows (and, in one instance, highlighted by a black box). Homochiral rows (***P***) are marked with blue arrows, while single racemic rows (***MP***) are indicated by black arrows. (E) STM image (30 nm × 30 nm, *I* = 2 nA, *U* = 100 mV) showing a domain that appears mirrored with respect to the structure in (B), partially superimposed with molecular models of both enantiomers and color coded with blue and red dots. One adsorbate lattice vector, placed over a homochiral dimer row, is indicated by a white arrow. Black arrows indicate an angle of 120°. The unit cell is outlined by a white parallelogram. A homochiral dimer row of (*M*) enantiomers is indicated by a semitransparent red line. The inset (8.6 nm × 5.2 nm) shows a magnified area (×2) of the upper left corner. (F) Structural models of the molecular arrangements in both mirror domains. Unit cells are indicated by black parallelograms, and the enantiomeric composition of the rows is denoted at their termini. Colored arrows correlate the models with STM images of (D) and (E).

Upon approaching monolayer saturation (~1.0 ML), the surface undergoes a complete densification transition. The Ag(111) terraces become fully covered by compact, well‐ordered islands (Figure 5A). In contrast to the low‐coverage regime, the porous honeycomb lattice is no longer observed. Instead, the molecules adopt a close‐packed arrangement. Comparison of the adsorbate lattice vectors (black arrows in Figure 5B) with the high‐symmetry directions of the Ag(111) surface (small white arrows) reveals an oblique alignment, suggesting the presence of mirror domains. High‐resolution STM images acquired in constant‐current mode (Figure 5B) reveal in part intramolecular contrast, enabling direct discrimination between the two enantiomers based on a brighter lobe at one terminus and a comma‐like molecular shape with a slightly wider end corresponding to the two CN groups. These features were used to assign the handedness.

Overlay of optimized molecular models onto the STM data (Figure 5C) confirms that both (*P*)*‐* and (*M*)*‐*diCN[4]H are incorporated within the same lattice. However, the distribution of enantiomers is not random. Structural analysis shows that the monolayer is composed of alternating racemic double rows and homochiral dimer rows. In Figure 5C, racemic double rows (***DR‐MP***) are highlighted by gray semitransparent boxes and indicated by white arrows, while homochiral dimer rows (***P***) are marked by blue semitransparent lines; a corresponding unit cell is outlined by a white rhombus. Within this domain, the local enantiomeric ratio amounts to 2:1. A color‐coded analysis (Figure 5D) also demonstrates the presence of systematic sequences with a clear local enantiomeric imbalance. Besides the alternating racemic double rows and homochiral dimer rows, there are also racemic single rows identified (***MP***, marked by black arrows). An area of alternating (***MP***) and (***P***) rows displays then an enantiomeric ratio of 3:1.

Conservation of the global racemic composition requires the presence of complementary domains elsewhere on the surface that exhibit the inverse enantiomeric excess. Figure 5E shows such a domain, displaying the opposite enantiomeric excess compared to that in Figure 5B. Because of the limited submolecular contrast, direct assignment of handedness is not feasible. Therefore, we first verified that this domain is the mirror counterpart of the one shown in Figure 5B. As the angle between the two homochiral dimer rows (indicated by long white arrows in Figures 5B and 5E) deviates from 120° (marked by short black arrows), a rotational domain relationship can be excluded, thereby confirming the mirror‐domain relationship. Additionally, structural motifs such as double rows and single dimer rows can be identified in parts of the image, enabling partial assignment of handedness. A structural model of the molecular arrangements in both mirror domains, including their unit cells, is presented in Figure 5F [50].

The variation in periodicity between enantiopure, racemic single, and racemic double rows may arise from strain relief associated with increasing lattice mismatch between the molecular two‐dimensional crystal and the underlying Ag surface. This reflects a delicate balance between preferred adsorption sites and lateral intermolecular interactions, such as hydrogen bonding involving the polar CN groups and repulsive forces due to dense packing, which may promote both structural rearrangements and local enantiomeric imbalance. Moreover, the low enantiomerization barrier of [4]helicene (3.5–4.4 kcal/mol) [49, 51] enables rapid interconversion between enantiomers at deposition temperature, potentially introducing an additional entropic contribution that influences the observed stereochemical distribution.

Importantly, the dense phase at monolayer saturation coverage does not correspond to a racemic solid solution. In a solid solution, the two enantiomers would occupy equivalent lattice sites in a statistical manner [52]. Here, instead, the enantiomers follow a regular, nonrandom pattern within the ordered lattice. The phase thus retains long‐range positional order while exhibiting a compositionally biased arrangement of handedness. Such enantiomerically unbalanced yet structurally ordered two‐dimensional crystallization is uncommon [52, 53], and it highlights the delicate interplay between chiral recognition and packing constraints at surfaces.

## 4 Conclusions

We have demonstrated that racemic diCN[4]H undergoes coverage‐dependent self‐assembly on Ag(111), evolving from spontaneous chiral resolution at low coverage to a dense, mixed phase at monolayer saturation. At submonolayer coverage, homochiral Kagomé domains form via energetically favored homochiral dimers, consistent with two‐dimensional conglomerate crystallization.

Upon densification, the porous network transforms into compact, substrate‐aligned domains incorporating both enantiomers. Their distribution is regular yet enantiomerically unbalanced, excluding a racemic solid solution and instead indicating a structurally ordered mixed phase. The observed densification therefore marks a transition from two‐dimensional conglomerate crystallization at low coverage, characterized by homochiral Kagomé domains, to a compact mixed phase at saturation in which maximal packing density necessitates deviation from strict homochirality. This coverage‐dependent evolution underscores how subtle differences in intermolecular interactions and steric demands govern chiral symmetry breaking and reorganization in surface‐confined molecular assemblies.

## Funding

This work was supported by Grantová Agentura České Republiky (24‐11064S) and Schweizerischer Nationalfonds zur Förderung der Wissenschaftlichen Forschung (212167, 173720, 182082, 202775, 221265).

## Data Availability

The data that support the findings of this study are available from the corresponding author upon reasonable request.
